# Comparison of clinical severity, genotype and toxin gene expression of binary toxin-producing *Clostridioides difficile* clinical isolates in Japan

**DOI:** 10.1099/acmi.0.000362

**Published:** 2022-10-20

**Authors:** Asami Matsumoto, Yuka Yamagishi, Kentaro Miyamoto, Seiya Higashi, Kentaro Oka, Motomichi Takahashi, Hiroshige Mikamo

**Affiliations:** ^1^​ Department of Clinical Infectious Diseases, Aichi Medical University, 1-1, Yazakokarimata, Nagakute City, Aichi 480-1195, Japan; ^2^​ R&D Division, Miyarisan Pharmaceutical Co., Ltd., 2-22-9, Toro-cho, Kita-ku, Saitama 331-0804, Japan; ^3^​ Department of Clinical Infectious Diseases, Kochi Medical School, 185-1, Kohasu, Oko-cho, Nankoku City, Kochi 783-8505, Japan; ^4^​ Department of Clinical Infectious Diseases, Aichi Medical University Graduate School of Medicine, 1-1, Yazakokarimata, Nagakute City, Aichi, 480-1195, Japan

**Keywords:** *Clostridioides difficile*, bacterial toxin, disease severity, cytotoxicity test, gene expression

## Abstract

The emerging *Clostridioides difficile* strain BI/NAP1/027 has been reported to be associated with more severe clinical symptoms and higher mortality rates, thought in part due to production of a novel binary toxin alongside conventional A and B toxins. However, recent studies suggest that this may not always be the case. Therefore, the purpose of this report was to investigate the correlation between clinical severity and microbiological characteristics of CDT-producing *C. difficile* isolates in Japan. Eight Japanese isolates of CDT producing *C. difficile* were investigated using genotyping, cytotoxic activity assays and toxin gene expression. Correlation with clinical severity was performed retrospectively using the patient record. Three of eight patients were assessed as having severe *C. difficile* infection (CDI). PCR ribotyping resolved six ribotypes including ribotype 027. No specific genes were identified determining severe compared with non-severe cases. Positive correlation of expression levels of *tcdA*, *tcdB* and *cdtB* were observed although these expression levels were not correlated with cytotoxicity. CDI severity index neither correlated with toxin gene expression level nor cytotoxicity. These data indicate that the possession of the CDT gene and toxin gene expression levels may not relate to *C. difficile* cytotoxicity or clinical severity.

## Introduction

*Clostridioides difficile* is well recognized as the leading cause of a spectrum of diseases from pseudomembranous colitis to *C. difficile *-associated diarrhoea, having a significant impact in both healthcare and community settings. The primary cause of *C. difficile* infection (CDI) is disruption of the gut microbiome by antibiotics used for treating other infections [1]. The two well characterized virulence factors are the exotoxins, Toxin A (an enterotoxin encoded by *tcdA*) and Toxin B (a cytotoxin encoded by *tcdB*). These toxins are likely to act by inactivation of Rho-family GTPases, leading to disruption of the actin cytoskeleton, cell-rounding, and apoptosis [2]. The cell death that a toxin causes leads to destruction of cellular tight junctions, which increases intestinal membrane permeability and promotes inflammation [3]. In addition to these two major toxins, some strains of *C. difficile* produce a third toxin called binary-toxin (CDT; *C. difficile* transferase), which may also be involved in the disease process. CDT is a binary actin-ADP-ribosylating toxin that catalyses irreversible ADP ribosylation of monomeric actin, resulting in disruption to the host cell cytoskeleton. Strains capable of producing CDT have been associated with more severe disease in humans. However, the role of this toxin in disease is still unclear [4].

Epidemiologically, from 2002 to 2003, an emerging hyper-virulent CDT-producing *C difficile* strain called BI/NAP1/027 (PCR ribotype 027) has been reported in North America and Europe causing severe CDI [5, 6]. In a Canadian study, *C. difficile* PCR ribotype 027 increased in incidence by four times in 2003 with three times higher mortality within 30 days compared to 1991 [7, 8]. In addition, the increasing CDI mortality from 5.7 per million population in 1999 to 23.7 per million in 2004 in the United States may correlate to increasing prevalence of PCR ribotype 027 [9, 10]. These epidemic strains are also identified in outbreaks in European countries [11]. In addition, PCR ribotype 078, a second CDT producing hyper-virulent ribotype was recently isolated from both human and pigs [12].

On the other hand, both PCR ribotype 027 and 078 have only been observed in sporadic cases in Japan, unlike in Europe and North America. The variant toxin A-negative/toxin B-positive (A-B+) PCR ribotypes 017 and 018 *C *. *difficile* strains are the major ribotype with a relatively low incidence of CDT producers in the Asia region [13].

From a clinical point of view, epidemiological and microbiological studies suggest that CDT producing strains may result in more severe CDI compared to non-CDT producing *C. difficile *. However, conflicting results have been obtained, with Miller *et al*. [14] reporting that clinical symptoms of patients with CDT positive PCR ribotypes were more severe than that of non-CDT producers, whereas Walker *et al*. [15] reported that the clinical symptoms were not correlated with CDT positivity.

Therefore, the aim of this study was to examine the correlation of clinical severity with microbiological differences including genotype, cytotoxic activities and gene expression among CDT-producing *C. difficile* strains isolated from Japanese CDI patients.

## Methods

### Patients

In this study, we selected eight inpatients diagnosed with CDI with CDT-producing *Clostridioides difficile* strains between 2009 and 2013 in Aichi Medical University Hospital, Aichi, Japan. Eight *C. difficile* strains were isolated from each patient as single strains. Written informed consent was obtained from all participants. The study protocol was approved by the Institutional Review Board of Aichi Medical University Hospital (No. 14–079).

### *Clostridioides difficile* strains

Five *C. difficile* ATCC strains (43593: ribotype 060, *tcdA* ^-^, *tcdA* ^-^, *cdtB* ^-^, 700057: ribotype 038, *tcdA* ^-^, *tcdA* ^-^, *cdtB* ^-^, 9689: ribotype 001, *tcdA* ^+^, *tcdB* ^+^, *cdtB* ^-^, BAA-1870: ribotype 027, *tcdA* ^+^, *tcdA* ^+^, *cdtB* ^+^, BAA-1875: ribotype 078, *tcdA* ^+^, *tcdA* ^+^, *cdtB* ^+^) were obtained from the American Type Culture Collection (Manassas, VA, USA) and used as reference strains.

*C. difficile* strains were plated on Gifu anaerobic medium (GAM) agar (Nissui Pharmaceuticals Co. Ltd., Tokyo, Japan) and grown at 37 °C under anaerobic conditions (80 % N_2_, 10 % CO_2_, and 10 % H_2_) in an anaerobic chamber for 24 h.

### Assessment of clinical severity

For clinical severity assessment, diarrhoea episodes, duration of hospitalization, age, body temperature, blood albumin level and white blood cell (WBC) counts, pseudomembranous colitis and ICU management were retrospectively obtained using the patient record. Clinical severity scores were then calculated using the Modified University of Illinois index [16]. One point was assigned for each of the following four risk factors; age > 60 years, fever (body temperature > 38.3 °C), hypoalbuminemia (serum albumin level ≤ 2.5 mg dl^−1^) and WBC count >15,000 cells mm^−3^. Furthermore, when infected patients demonstrated pseudomembranous enteritis or were admitted to the ICU, two points were added. Patients with more than two points were defined as severe CDI.

### DNA extraction, library preparation and Whole Genome Sequence (WGS)

*C. difficile* isolates were grown in GAM broth (Nissui Pharmaceuticals Co. Ltd., Tokyo, Japan) at 37 °C in an anaerobic chamber (80 % N_2_, 10 % CO_2_, and 10 % H_2_) for 24 h. The bacteria were collected by centrifugation at 5,000 ***g*** for 10 min and the pellets were suspended in 10 mM Tris-HCl and 10 mM EDTA buffer (pH 8.0) and incubated with lysozyme (Sigma-Aldrich, Inc., St. Louis, MO, USA. final concentration: 15 mg ml^−1^) at 37 °C for 1 h. A purified achromopeptidase (Wako Pure Chemical Industries, Ltd., Osaka, Japan) was added (final concentration: 2,000 U ml^−1^) and further incubated at 37 °C for another 30 min. SDS (final concentration: 1 %) and proteinase K (Merck KGaA, Darmstadt, Germany) were added (final concentration: 1 mg ml^−1^) to the suspension, mixed and incubated at 55 °C for 1 h. DNA samples were purified by treatment with RNase A (Wako Pure Chemical Industries, Ltd., Osaka, Japan), followed by precipitation with phenol/chloroform/isoamyl alcohol (Nippon Gene Co., Ltd., Tokyo, Japan) and 20 % PEG solution (PEG6000 in 2.5 M NaCl). DNA was pelleted by centrifugation, rinsed with 75 % ethanol, and dissolved in TE buffer.

Genomic DNA was processed using a focused-ultrasonicator (Covaris, Inc. Woburn, MA, USA), and then extra fragments were removed in a double size selection by using SPRIselect (Beckman Coulter, Inc., Brea, CA, USA).

The bacterial DNA libraries were prepared using the NEBNext DNA Library Prep master mix set for Illumina (New England Biolabs, Inc., Ipswich, MA, USA). The final libraries were analysed on a 2100 Bioanalyzer using a DNA chip (Agilent Technologies, Inc., Waldbronn, Germany). WGS were obtained using an Illumina MiSeq sequencer (Illumina, Inc., San Diego, CA, USA), with sequencing runs for paired-end sequences.

WGS data are available from the DNA DataBank of Japan (DDBJ) Sequence Read Archive (DRA) under the accession number DRA011384.

### Data analysis and genetic characterization

The sequence reads were assembled using Platanus assembler v 1.2.4 [17]. and annotated with PROKKA v 1.12 [18]. The multilocus sequence typing (MLST) was performed by PubMLST [19]. The multiple sequence alignments and construction of the phylogenetic tree were performed using the Mauve software [20] and FigTree v1.4.2. The predicted genes in the WGS were clustered by using the CD-hit programme [21] with the parameters c=0.7 and aL=0.7. The representative sequences of each cluster detected in CD-hit were subjected to BLASTP searches with an E value cut-off of 1E-10 against the Virulence Factors Database (VFDB) [22] for determining the virulence genes and the Comprehensive Antibiotic Resistance Database (CARD) [23] for determining the antibiotic resistance genes.

### PCR ribotyping

PCR ribotyping of *C. difficile* isolates was performed by the method described previously [24]. Briefly, the amplified PCR products were concentrated to a final volume of approximately 10 µl by heating at 75 °C for 90 to 120 min before electrophoresis in 3 % Metaphor agarose (Lonza Rockland, Inc., Basel, Switzerland) at a constant voltage of 100 V for 4 h. Isolates with patterns differing by one or more bands were assigned to different PCR ribotypes, and the difference in faint bands was ignored.

### Toxin gene detection and in-frame deletion typing of *tcdC*

Toxin gene possession and in-frame deletion of *tcdC* were analysed by multiplex PCR as previously described by others [25, 26]. This method allows the simultaneous identification of the toxin genes *tcdA*, *tcdB*, *cdtA*, and *cdtB* combined with an approximate determination of the in-frame *tcdC* deletion size.

### Quantification of toxin gene expression

Toxin gene expression levels (*tcdA*, *tcdB*, *cdtB*) of *C. difficile* strains were quantified by an RT-qPCR method using specific primer sets using a method described by others with slight modifications [27]. Primers for *tcdA* as previous described by Woo *et al*. [27], and primers for *tcdB* and *cdtB* as described by Wroblewski *et al*. were used [28]. Briefly, *C. difficile* isolates were grown in GAM broth (Nissui Pharmaceuticals Co. Ltd., Tokyo, Japan) at 37 °C in an anaerobic chamber (80 % N_2_, 10 % CO_2_, and 10 % H_2_) for 24 h. The culture supernatants of *C. difficile* were used for RNA extraction. Total RNA was isolated by using TRIZOL reagent according to the manufacturer’s instructions (Thermo Fisher Scientific, Inc., MA, USA). The cDNA was obtained from messenger RNA with a Primescript RT reagent kit according to the manufacturer’s instructions (Takara Bio, Inc., Otsu, Japan). RT-qPCR was carried out using SYBR *Premix Ex Taq* (Takara Bio, Inc., Otsu, Japan) in the Thermal Cycler Dice Real Time System II (Takara Bio, Inc., Otsu, Japan). Results were analysed using Thermal Cycler Dice Real Time System II Software (version 5.00). The expression levels of all toxin genes tested in each isolate were normalized using the *C. difficile* housekeeping gene *rpoA* as a reference [29].

### Cytotoxicity assay

Cytotoxicity was determined by the method described previously [24]. Briefly, in 96-well microplates, African green monkey kidney (Vero) cells were grown in Eagle’s minimum essential medium (MEM; Sigma-Aldrich, Inc., St. Louis, MO, USA) supplemented with 10 % fetal calf serum (Sigma-Aldrich, Inc., St. Louis, MO, USA) in a 5 % CO_2_ incubator and the cell culture medium was removed and replaced with 100 µl of fresh sustenance medium (Eagle’s MEM containing 1 % fetal calf serum) before assay. *C. difficile* isolates were grown in GAM broth (Nissui Pharmaceuticals Co. Ltd., Tokyo, Japan) at 37 °C in an anaerobic chamber (80 % N_2_, 10 % CO_2_, and 10 % H_2_). The culture supernatants of *C. difficile* were prepared by centrifuging at 5,000 ***g*** for 10 min and filtering to sterilize. The *C. difficile* supernatants were subjected to two-fold serial dilution with sustenance medium. The cell culture medium was then removed and replaced with 100 µl of each diluted sample. The cytotoxicity of the sample was determined to be the highest dilution resulting in 100 % cell rounding after incubation for 24 h.

### Correlational analysis

Non-parametric Spearman’s rank correlations were used for the correlation analysis between patient information, clinical severity, toxin gene expression levels and cytotoxicity.

## Results

### Clinical severity

We investigated the clinical severity of the eight patients who were diagnosed with CDI with CDT producing *C. difficile* in the hospital. The median number of diarrhoea episodes was three times (0–10 times) and median duration of hospitalization was 54 days (13–142 days) (Table 1). Median age was 67 years (3–74 years). Median values for biological data were 3.3 mg dl^−1^ (1.8–4.1 mg dl^−1^) for serum albumin concentration and 13,250 cells mm^−3^ (2,400–31, 300 cells mm^−3^) for WBC. Three of the eight patients (37.5 %; Pat. No. 2, 3 and 7) were classified as severe CDI (All patients were score 2). The remaining five patients (62.5 %; Pat. No. 1, 4, 5, 6 and 8) were classified as not severe (Pat. No. 1, 4, 5 and 8 scored one and Pat. No. 6 scored 0). No patients developed pseudomembranous colitis or were admitted to the ICU.

**Table 1.** Clinical severity assessment of eight inpatients

Pat. No.	Strain	Diarrhoea episodes	Duration of hospitalization (days)	Laboratory test values for severity score (Modified University of Illinois) and criteria						Severity score
				Age	Body temp.	Alb level	WBC count	Pseudomembranous colitis	ICU management	
				>60 years old	>38.3 °C	<2.5 g dl^−1^	>15,000 counts/μl			Severe ≥2
1	CD52	10	13	70y8m	37.0	4.0	14,100	ns	ns	1
2	CD110	10	59	67y9m	36.8	1.8	11,400	ns	ns	2
3	CD253	0	31	67y8m	36.0	4.1	30,800	ns	ns	2
4	CD329	0	64	67y7m	37.2	2.9	3,500	ns	ns	1
5	CD374	4	17	74y4m	37.1	3.6	12,400	ns	ns	1
6	CD391	2	142	50y1m	36.0	3.4	2,400	ns	ns	0
7	CD546	8	53	70y4m	38.2	3.2	31,300	ns	ns	2
8	CD602	0	55	3y11m	38.0	2.6	16,400	ns	ns	1

The severity score was evaluated using Modified University of Illinois index. The evaluation items were age (> 60 years old), temperature (> 38.3 °C), albumin level (< 2.5 g dl^−1^) and white blood cell (WBC) count (> 15, 000 counts μl^−1^) were one point. Two points were added for pseudomembranous colitis and ICU admission. This index indicated severe CD infection with scores of 2 or more.

Alb, albumin; m, month; NI, No Information; NS, No Symptoms; Pat No., Patient number; WBC, white blood cell; y, years.

### PCR ribotyping patterns of *C. difficile*

**Fig. 1.**
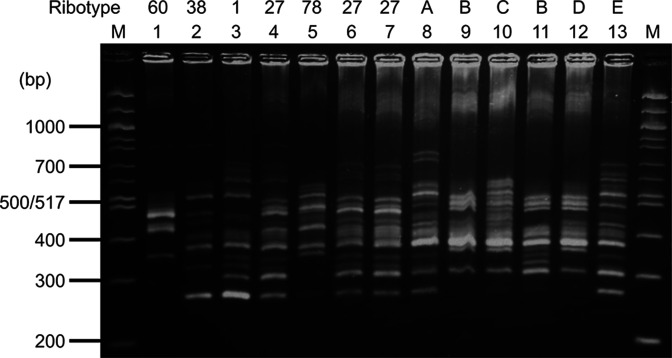
PCR ribotyping pattern of *C. difficile *. PCR ribotyping pattern analysis was performed. Eight *C. difficile* isolates are represented in lanes 6 to 13 (CD52, CD110, CD253, CD329, CD374, CD391, CD546 and CD602, respectively). ATCC strains in lanes 1 to 5 were used as control strains (ATCC 43593 (ribotype 060, *tcdA* ^-^, *tcdA* ^-^, *cdtB* ^-^), ATCC 700057 (ribotype 038, *tcdA* ^-^, *tcdA* ^-^, *cdtB* ^-^), ATCC 9689 (ribotype 001, *tcdA* ^+^, *tcdB* ^+^, *cdtB* ^-^), ATCC BAA-1870 (ribotype 027, *tcdA* ^+^, *tcdA* ^+^, *cdtB* ^+^), ATCC BAA-1875 (ribotype 078, *tcdA* ^+^, *tcdA* ^+^, *cdtB* ^+^)). M indicates 100 bp ladder size marker. Eight isolates were classified in six types, and two isolates showed PCR ribotype 027.

PCR ribotyping was performed to determine the genotype of *C. difficile* clinical isolates. Five ATCC strains were used as reference strains of known ribotypes. All the eight clinical isolates resolved into six PCR ribotypes (ribotype 027 and A to E) (Fig. 1). Two strains were assigned to ribotype 027 and six strains demonstrated different ribotypes to reference strains (ribotype 060, 038, 001, 027 or 078).

### Toxin gene profiles and *tcdC* in-frame deletion typing of *C. difficile*

**Fig. 2.**
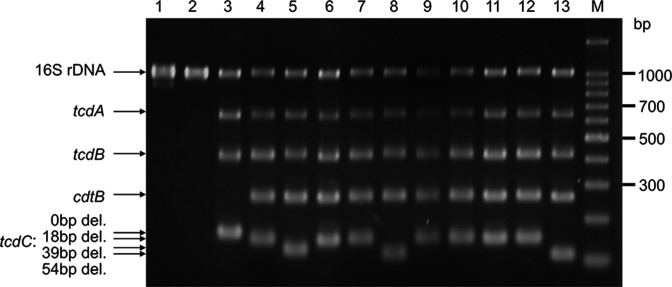
. *C. difficile* toxin gene detection and in-frame deletion typing of *tcdC*. PCR assay of eight clinical isolates (lanes 6 to 13; CD52, CD110, CD253, CD329, CD374, CD391, CD546 and CD602, respectively.) and five ATCC strains (lanes 1 to 5; ATCC 43593, ATCC 700057, ATCC 9689, ATCC BAA-1870, ATCC BAA-1875, respectively.) demonstrating toxin gene profiles of *tcdA*, *tcdB*, and *cdtB*, presence of a *tcdC* in-frame deletion (del.) of 0, 18, 39, or 54 bp and internal PCR control directed toward 16S rDNA genes. M indicates 100 bp ladder size marker.

To confirm the correlation between severity and toxin gene profiles, especially deletion in *tcdC*, we investigated the toxinotype and *tcdC* in-frame deletion types of the clinical isolates. All eight strains were *tcdA*, *tcdB* and *cdtB* positive (Fig. 2). Six strains had 18 bp in-frame deletions and two had 54 bp in-frame deletions in *tcdC*.

### *C. difficile* MLST

**Fig. 3.**
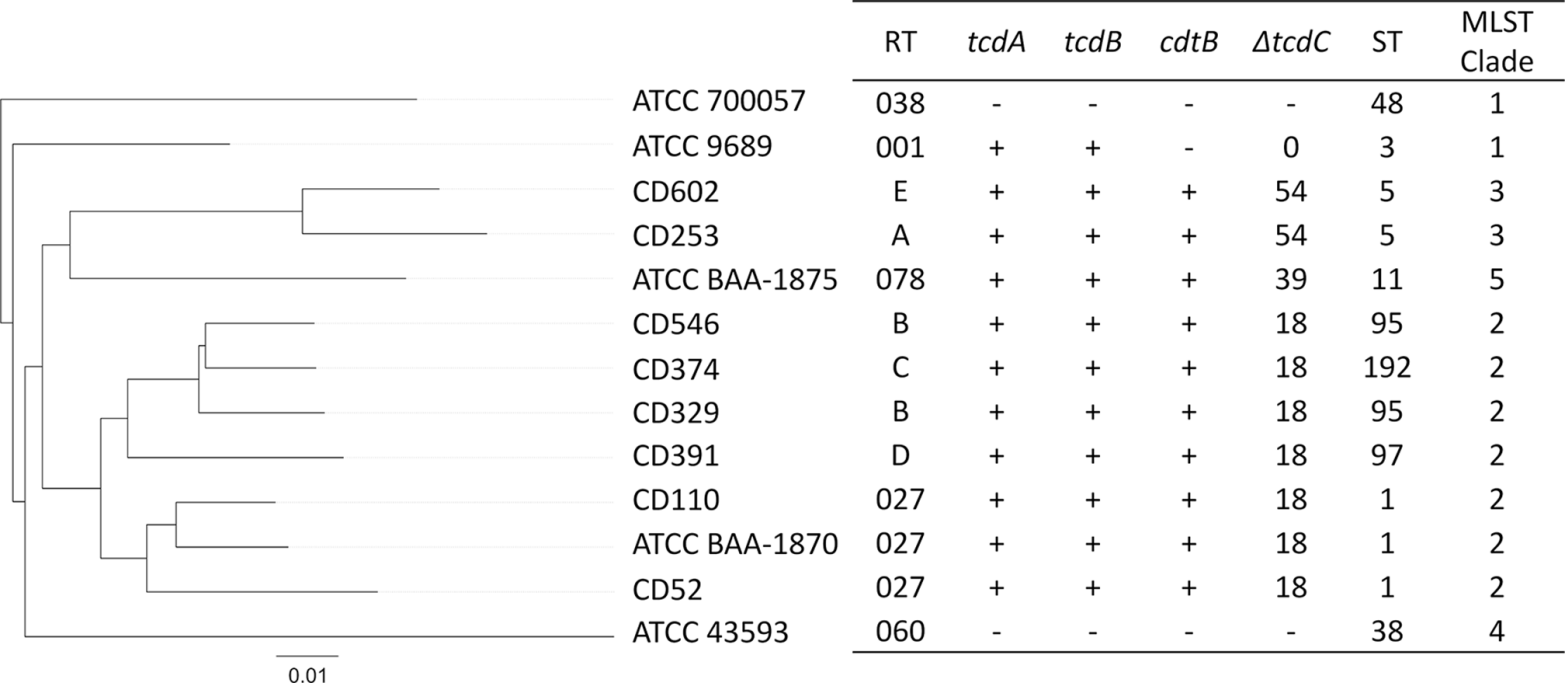
Phylogenetic and MLST analysis of *C. difficile *. Whole genome phylogeny of the genomes of eight clinical isolates and five ATCC strains. Scale bars for the top and bottom sections indicate distances in fractional change in nucleotide sequence. STs and MLST clades were detected using PubMLST.

MLST revealed five different sequence types (STs) and two different MLST clades were found among the eight isolates (Fig. 3). Two isolates belonged to ST 1 (CD52 and CD110), two isolates belonged to ST5 (CD253 and CD602), two isolates belonged to ST95 (CD329 and CD546), one isolate belonged to ST97 (CD391) and one isolate belonged to ST192. Six isolates belonged to Clade2 (CD52, CD110, CD329, CD374, CD391 and CD546) and two isolates belonged to Clade3 (CD253 and CD602).

### Phylogenetic relationships among isolates

The phylogenic tree was drawn by using the WGS of eight clinical isolates and five ATCC strains. Thirteen strains were classified in four groups (Fig. 3). Group 1 contains one strain isolated from a severe CDI case (CD253), one strain isolated from a non-severe CDI case and ATCC BAA-1875. Group 2 contains one strain isolated from a severe CDI case (CD546) and three strains isolated from non-severe CDI cases. Group 3 contains the CD52 strain isolated from a non-severe case and the CD110 strain isolated from a severe case and also contains ATCC BAA-1870. The other strains (ATCC 43593, ATCC 9689 and ATCC 700057) were classified into Group 4.

### Gene prediction and annotation

To determine any correlation of genotype with severity of CDI, WGS were clustered by using the CD-hit programme. A total of 5,419 genes were predicted in CD-hit. Of these predicted genes, 293 were detected from isolates which occurred in severe CDI (CD110, CD253 and CD546) and 5,126 were detected from isolates which were common to non-severe and severe CDI. The representative sequences of each cluster detected in CD-hit were subjected to BLASTP against the VFDB and CARD. Fourteen virulence genes and ten antimicrobial resistance genes were detected in severe CDI isolates (Tables 2 and 3).

**Table 2.** Analysis of virulence genes in *C. difficile* strains

VFDB accession no.	Virulence gene	Product	CD110	CD253	CD546
VFG038722(gb|YP_855928)	-	CobQ/CobB/MinD/ParA family protein	−	+	−
VFG037274(gb|YP_001847233)	*barA*	Siderophore efflux system of the ABC superfamily	+	−	−
VFG038234(gb|YP_001845365)	*bfmS*	Signal transduction histidine kinase	+	−	−
VFG000421(gb|NP_395427.1)	*caf1R*	F1 operon positive regulatory protein	−	+	−
VFG049159(gb|YP_006635485.1)	*clbM*	Precolibactin export MATE transporter ClbM	−	+	−
VFG005776(gb|NP_687685)	*cylA*	ABC (ATP-binding cassette) transporter CylA	−	+	−
VFG045470(gb|AAM75247)	*cylR2*	Cytolysin regulator R2	−	−	+
VFG038532(gb|YP_855994)	*flpF*	Traffic ATPase	−	+	−
VFG000070(gb|NP_464110)	*iap/cwhA*	P60 extracellular protein, invasion associated protein Iap	−	+	−
VFG000574(gb|NP_462662)	*mgtB*	Mg^2+^ transport protein	−	−	+
VFG000169(gb|NP_252919)	*pchC*	Pyochelin biosynthetic protein PchC	−	+	−
VFG001386(gb|NP_215271)	*phoP*	Possible two component system response transcriptional positive regulator PhoP	−	+	−
VFG009810(gb|NP_215272)	*phoR*	Possible two component system response sensor kinase membrane associated PhoR	−	+	+
VFG039025(gb|YP_505894)	*virD4*	Type IV secretion system component VirD4	−	+	−

The virulence genes were identified in isolates derived from three patients with severe CDI (CD110, CD253 and CD546) using the CD-hit programme and the Virulence Factors Database. Fourteen virulence genes were detected from three patients.

**Table 3.** Analysis of antimicrobial resistance genes in *C. difficile* strains

CARD accession no,	Antimicrobial resistance gene	Gene family	CD110	CD253	CD546
ARO:3 002 630	*ant(9)-Ia*	ANT(9)	−	+	−
ARO:3 002 639	*aph(3'')-Ib*	APH(3'')	−	+	−
ARO:3 003 835	*cdeA*	Multidrug and toxic compound extrusion (MATE) transporter	−	+	−
ARO:3 000 504	*golS*	Resistance-nodulation-cell division (RND) antibiotic efflux pump	−	+	+
ARO:3 003 549	*mdtO*	Major facilitator superfamily (MFS) antibiotic efflux pump	−	+	−
ARO:3 000 823	*ramA*	General Bacterial Porin with reduced permeability to beta-lactams; resistance-nodulation-cell division (RND) antibiotic efflux pump	−	+	−
ARO:3 003 794	*walK*	Daptomycin resistant *walK*	−	+	+
ARO:3 000 168	*tet(D*)	Major facilitator superfamily (MFS) antibiotic efflux pump	−	+	−
ARO:3 002 919	*vanRA*	Glycopeptide resistance gene cluster; *vanR*	−	+	−
ARO:3 002 934	*vanSD*	Glycopeptide resistance gene cluster; *vanS*	+	−	−

The antimicrobial resistance genes were identified in isolates derived from three patients with severe CDI (CD110, CD253 and CD546) using the CD-hit programme and the Comprehensive Antibiotic Resistance Database. Ten antimicrobial resistance genes were detected from three patients.

### Toxin gene expression and cytotoxicity

Toxin gene expression as a ratio compared to *rpoA* was different between *C. difficile* isolates (*tcdA*/*rpoA*, 0.24±0.05–0.73±0.03; *tcdB*/*rpoA*, 0.08±0.02–0.80±0.10; *cdtB*/*rpoA*, 0.30±0.09–0.80±0.15) (Table 4). The *tcdA* expression levels in one isolate was high (0.7 <; CD52), three isolates were moderate (0.4–0.6; CD110, CD374 and CD391), whereas other strains (0.4 >; CD253, CD329, CD546 and CD602) demonstrated low levels. The *tcdB* expression levels in one isolate was high (0.7 <; CD52), two isolates were moderate (0.4–0.6; CD110 and CD391), whereas other strains (0.4 >; CD253, CD329, CD374, CD546, CD602) demonstrated low levels. The *cdtB* expression levels in three isolates was high (0.7 <; CD52, CD374 and CD391), one isolate was moderate (0.4–0.6; CD110), whereas other strains (0.4 >; CD253, CD329, CD546, CD602) demonstrated low levels. The results of the cytotoxicity assay demonstrated five isolates with high toxin titre (2^7^-2^9^; CD52, CD110, CD374, CD391 and CD546), two moderate (toxin titre 2^5^–2^6^; CD253, CD329) and one low (toxin titre 2^4^; CD602).

**Table 4.** Toxin gene expression and cytotoxicity of *C. difficile* isolates

Pat. no.	Strain no.	Toxin gene expression			Cytotoxicity
		*tcdA*/*rpoA*	*tcdB*/*rpoA*	*cdtB*/*rpoA*	
	ATCC 43593	nd	nd	nd	<2^1^
	ATCC 700057	nd	nd	nd	<2^1^
	ATCC 9689	0.91±0.10	1.08±0.10	nd	2^10^
	ATCC BAA-1870	0.99±0.09	1.00±0.11	1.10±0.03	2^9^
	ATCC BAA-1875	0.55±0.01	0.64±0.10	0.47±0.02	2^11^
1	CD52	0.73±0.03	0.80±0.10	0.80±0.15	2^8^
2	CD110	0.55±0.07	0.58±0.03	0.59±0.07	2^9^
3	CD253	0.29±0.04	0.35±0.02	0.33±0.06	2^5^
4	CD329	0.25±0.05	0.26±0.02	0.32±0.06	2^6^
5	CD374	0.51±0.03	0.08±0.02	0.72±0.14	2^8^
6	CD391	0.59±0.05	0.68±0.01	0.72±0.14	2^7^
7	CD546	0.37±0.02	0.38±0.03	0.45±0.03	2^9^
8	CD602	0.24±0.05	0.34±0.08	0.30±0.09	2^4^

Underlining differentiates reference strains and clinical isolates. Means and standard deviations of relative toxin gene (*tcdA*, *tcdB* and *cdtB*) expression from triplicate assays are shown. Each toxin gene expression was normalized using *rpoA* as a reference. Cytotoxicity is also expressed as a mean of assays done in triplicate.

ND, not detected; Pat No., patient number.

### Correlation between clinical severity and microbiological characteristics

**Fig. 4.**
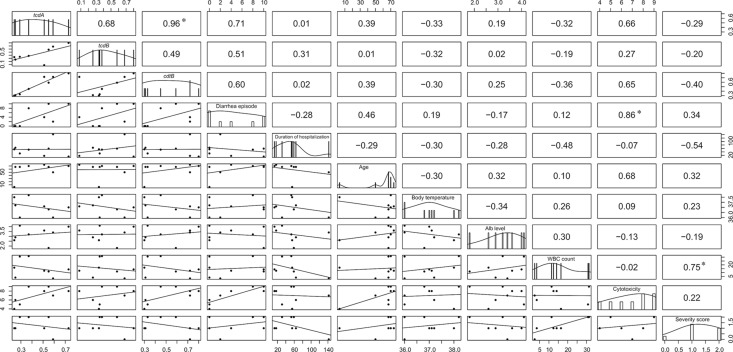
Matrix of scatter plots of clinical and microbiological data. A correlation of toxin genes, diarrhoea episodes, duration of hospitalization, age, body temperature, Alb level, WBC count, cytotoxicity and severity score were calculated by Spearman’s rank correlation coefficient. The correlation coefficients and *p* value were calculated, and the correlation coefficient value is shown in the figure. Asterisks denote statistical significance (*P* < 0.05). The expression of toxin genes showed a correlative tendency. The number of diarrhoea episodes positively correlated with cytotoxicity. WBC count positively correlated with severity score.

To investigate the relationship between clinical severity and microbiological characteristics, we performed a correlation analysis using a non-parametric Spearman’s rank correlation. Correlations between expression levels of *tcdA*, *tcdB* and *cdtB* were observed except for one strain (strain CD374) (Fig. 4). There was no correlation between cytotoxicity and toxin gene expression levels (*tcdA*, *tcdB* and *cdtB*). No correlations were observed between severity score, toxin gene expression levels (*tcdA*, *tcdB* and *cdtB*) or cytotoxicity (*P* ≥ 0.05). However, the number of diarrhoea episodes was positively correlated with cytotoxicity. Furthermore, WBC count was positively correlated with severity score.

## Discussion

*Clostridioides difficile* is a spore-forming, anaerobic intestinal pathogen that causes severe diarrhoea that can lead to death. In 2011, *C. difficile* infected more than 500,000 people in the United States and killed more than 29,000 people. Moreover, *C. difficile* infection (CDI) is the most common healthcare-related infection in the USA, leading to increased healthcare costs. In recent years there have been few serious cases in Japan, but the prevalence of the CDI is increasing [30].

The relationship between virulence factors and clinical severity of patients with *C. difficile* infection has been reported previously but is still unclear. According to previous reports [16] and guidance for the diagnosis of CDI [31], major factors for assessment of clinical status include age, body temperature, albumin level and peripheral WBC counts. Age, albumin level and WBC usually correlate with patients with CDT positive *C. difficile* infection [15, 32]. However, our results showed that only three out of eight patients showed severe symptoms and the other five CDT positive CDI patients showed non-severe clinical symptoms (Table 1).

Our data corresponds to previous reports detailing cases which show non-severe infection by CDT producing strains [15]. Some inpatients had other conditions in addition to CDI, for example: acute pneumonia, ANCA-associated vasculitis, acute respiratory failure and renal failure. Previous studies reported that renal disease may increase the risk of severe CDI [33, 34]. In this study, two of three inpatients with severity score two also had renal failure again suggesting an association between severe CDI and renal failure. Two of the three inpatients categorized as severity score two were also diagnosed with respiratory distress syndrome, which has also been associated with severe CDI [35]. Together, these findings suggest that severe CDI is likely due to a combination of strain virulence and underlying patient factors.

The prevalence of CDI in children has increased in recent years over the past decade, with the BI/NAP1/027 strain being said to correspond to 10–19 % of the toxin-producing *C. difficile* in children with CDI [36]. In fact, patients under 10 years old accounted for around 60 % of the sample in the study by Mardaneh *et al*. assessing the detection of pathogenic toxin A/B-positive *C. difficile* strains among hospitalized patients with diarrhoea and gastroenteritis [37]. In this study, one sample was from an 3 year, 11 month old infant. It has been reported that nearly 50 % of infants carry *C. difficile* without symptoms of diarrhoea or necrotizing enterocolitis regardless of maternal acquisition, delivery method, or antibacterial treatment [38]. Infants have a high prevalence of *C. difficile* and are often asymptomatic even if *C. difficile* is colonized, but since an increase in incidence of CDI has also been reported, this is becoming of increasing relevance.

PCR ribotyping showed that all eight *C *. *difficile* strains were classified into six types with two of eight isolates assigned to ribotype 027 (Fig. 1). Several studies have demonstrated the molecular epidemiology of outbreaks caused by the BI/NAP1/027 strain in North America and Europe while there are few reports of epidemics of CDI by ribotype 027 in Japan [39]. Previous reports suggest an increased prevalence of ribotypes 018 and 017 in Asia, but we were not able to judge the data in this study [13].

Previous reports showed in-frame deletions of the *tcdA/tcdB* negative regulator *tcdC* in CDT producing strains and this is also seen in our isolates [40, 41]. However, although some isolates demonstrated a *tcdC* in-frame deletion, we did not observe an increase in clinical severity in these patients (Table 1, Fig. 2). This is contrary to reports suggesting that *tcdC* deletion causes severe disease and increased toxin production [42–44]. Our results instead support other studies suggesting that severe CDI is most likely caused by other factors and not solely determined by the *tcdC* in-frame deletion [42–44].

As a result of MLST analysis using PubMLST, eight isolates were classified in six MLST types and two clades (Fig. 3). CD52, CD110, CD329, CD374, CD391 and CD546 were classified in Clade2. RT244 (ST41) and RT176 (ST1) which cause severe CDI as well as the well-known hyper-virulent strain RT027 also belong to Clade2 [45]. In addition, CD253 and CD602 classified in Clade3 showed ST5 which was an ST type similar to RT023 detected in the EU [11]. CD329 and CD546 belong to ST95, and RT075 related to RT027 is also included in this ST type [46]. ST97 in which CD391 is classified also belongs to Clade2 with RT027 [47]. CD391 did not cause severe disease in this study and the patient recovered without antimicrobial treatment in the report from Sawabe *et al*. [48], suggesting that this ST is less likely to cause severe disease. CD374 showed ST192, and it was reported that ST192 was equivalent to RT080 in a Finnish study [49]. The results of this study suggest that being related to other more virulent strains in the same ST type or clade does not necessarily confer severe disease.

The phylogenetic tree of the total 13 strains consisting of eight clinical isolates and five reference strains, showed that the strains were classified in four clusters (Fig. 3). CD52 and CD110 which were RT027 were classified in a cluster with ATCC BAA-1870, but only CD110 caused severe CDI. This result was similar to that from the cluster containing CD329, CD374, CD391 and CD546 (CD329, CD374 and CD391 did not cause severe infection, CD546 was a severe case), and the cluster containing CD253, CD602 and ATCC BAA-1875 (RT078) (CD602 did not cause severe infection, CD253 was a severe case). These findings support previous reports such as by Walk *et al*. [15], suggesting that ribotypes and genotypes do not necessarily relate to disease severity.

Fourteen virulence genes and ten antimicrobial resistance genes were detected in three isolates causing severe disease (Tables 2 and 3). No one gene was common between all isolates suggesting that there is no simple relationship between any one locus and severity. CD253 demonstrated the largest number of virulence and resistance genes.

Though all clinical isolates were binary toxin producing strains, toxin gene expression and cytotoxicity differed between strains (Table 4). Several studies demonstrated that *C. difficile* ribotype 027 has increased toxin production [5, 29] and therefore, our result differed from previous reports.

In our correlation analysis, the expression of *tcdA* and *cdtB*, the number of diarrhoea episodes and cytotoxicity, WBC count and severity score were significantly correlated (Fig. 4). The expression levels of *tcdA* and *tcdB* and *tcdB* and *cdtB* were not significantly correlated but showed a correlative tendency. On the other hand, there was no correlation between severity, cytotoxicity, and expression of toxin genes and/or expression of toxin genes and cytotoxicity. These results support previous reports which demonstrated CDT positive *C. difficile* does not always cause severe disease [15, 39–41]. Our results support the hypothesis that hyper-virulent *C. difficile* strains such as ribotype 027 do not necessarily have an influence on clinical severity, expression of toxin genes and cytotoxicity.

Our results indicate that the possession of the CDT gene may not be directly related to high toxigenicity or clinical severity in Japanese CDI patients. Also, the in-frame deletion of the *tcdC* negative regulator of *tcdA* and *tcdB*, may also not correlate with high toxin production in *C. difficile *. Furthermore, toxin gene expression level and cytotoxicity may not be necessarily related to clinical symptoms, although it is possible that other medical conditions and co-morbidities affect the results of the severity assessment. Therefore, it is necessary to take care not to conclude whether disease is severe or non-severe only by confirming the ribotype, but to treat while considering the comorbidities of the patient and the characteristics of the strain.
